# Is Prolonged Copper Restriction Needed in Pediatric Wilson’s Disease?

**DOI:** 10.5152/tjg.2022.22216

**Published:** 2023-01-01

**Authors:** Hayriye Hizarcıoğlu-Gülşen, Pınar Şimşek Onat, Damla Yıldırım, Duygu Demirtaş, Meryem S. Boyraz, Mehmet A. Göktaş, Hülya Demir, Hasan Özen, İnci Nur Saltık-Temizel

**Affiliations:** 1 Department of Pediatric Gastroenterology, Hepatology and Nutrition, Hacettepe University Faculty of Medicine, Ankara, Turkey; 2 Department of Dietetics and Nutrition, İhsan Doğramacı Children’s Hospital, Hacettepe University, Ankara, Turkey; 3 Department of Pediatric Gastroenterology, Sağlık Bilimleri University, Van Training and Research Hospital, Van, Turkey; 4 Department of Pediatric Gastroenterology, Sağlık Bilimleri University, Başakşehir Çam ve Sakura City Hospital, İstanbul, Turkey

**Keywords:** Copper, diet therapy, hepatolenticular degeneration, malnutrition, nutritional status

## Abstract

**Background:**

Dietary copper restriction in Wilson’s disease is recommended mostly for 1 year or until showing normal liver enzymes. Little is known about the effect of long-term copper restriction on copper and nutritional status in the body. The relationship between daily copper consumption and serum and urine copper parameters, liver enzymes, and dietary contents was investigated.

**Methods:**

In this study, 32 pediatric Wilson’s disease patients who had been on treatment at least for 12 months were included. Clinical features, liver enzymes, serum total copper concentrations, non-ceruloplasmin bound copper concentrations, adjusted copper concentrations, 24-hour urine copper excretions, and macro- and micronutrient consumptions were analyzed.

**Results:**

In total, 27 patients reported following copper-restricted diets, while daily copper consumption was low only in 7 patients (21.9%). Total copper concentrations and non-ceruloplasmin-bound copper concentrations were low at 78.1% and 53.1%, respectively. All but one adjusted copper concentration were within normal limits. Total copper concentrations, adjusted copper concentration, and non-ceruloplasmin-bound copper concentrations correlated with each other but none correlated with urine copper excretions. Daily copper consumption was inversely correlated with total copper concentrations (*P* = .041, *r =* –0.363) but not correlated with non-ceruloplasmin-bound copper concentrations and adjusted copper concentrations. There was no relationship between liver enzymes and daily copper consumption and serum and urine copper parameters. High fat consumption with low fiber and vitamin B6 was more common in low daily copper consumption group (*P* = .033,* P* = .029, *P* = .007, respectively).

**Conclusions:**

Daily copper consumption may be the least effective or non-effective factor on liver enzymes in Wilson’s disease. Prolonged copper restriction may result in unintentional dietary imbalance. Avoidance of undernutrition and high-fat meals, as well as enrichment of the meals with vitamin B6 and fiber, should be encouraged during copper-restricted diets.

**Main Points**

- Dietary copper restriction is a supportive therapy in Wilson’s disease, but the duration and effects on liver enzymes are still questionable.
- Dietary copper may be the least effective or non-effective factor on elevated liver enzymes in Wilson’s disease.
- Prolonged copper restriction may cause avoidance of consuming protein and total calorie but high fat intake.
- During copper-restricted diet, preventing malnutrition and high-fat consumption along with consuming sufficient vitamin B6 and fiber should be guaranteed.

## Introduction

Copper is an essential trace element that acts as a cofactor for various metalloenzymes that participate in the respiratory chain, biosynthesis of neuroendocrine enzymes, heme metabolism, pigment and connective tissue formation, and body defense via free radicals.^1,2^ In physiological conditions, serum total copper concentration (TCC) is determined by the balance between intestinal absorption and biliary excretion.^3^

Wilson’s disease (WD) is characterized by copper accumulation primarily in the liver due to a mutation of the ATP7B gene that is responsible for biliary copper excretion from the hepatocyte and delivering copper to apoceruloplasmin to generate functional ceruloplasmin (holoceruloplasmin).^2,4^ Copper saturation in the liver causes cell injury, and subsequent release of non-ceruloplasmin bound-copper from the liver into the bloodstream results in extrahepatic copper accumulation in WD.^5,6^ Serum total copper level is not accepted as a diagnostic parameter because it may be low, normal, or high in WD.^7^ However, in most cases, except for severe hepatic injury, impaired gene function causes decreased serum ceruloplasmin and TCC but increased non-ceruloplasmin-bound copper concentration (NCBC) which is toxic for tissues.^2^ Increased 24-hour urine copper excretion (UCE), as well as high hepatic copper, is a valuable factor for WD diagnosis.^6^

Removal of toxic copper in the urine by chelating agents [D-penicillamine (DPA) and trientine] and blockage of intestinal copper absorption by zinc salts are the cornerstone of the WD treatment. Treatment compliance is indirectly monitored by UCE, serum copper levels, and liver enzymes.^6^ Dietary restriction of copper is recommended as a supportive therapy mostly until normalization of the liver enzymes^7^ or for 1 year,^8^ but the decision-making about the duration is still challenging, especially while managing patients with high liver enzymes. Besides, excessive and prolonged restriction of copper in diet may result in malnourishment as copper-rich foods are also rich in protein. However, the long-term effects of the copper-restricted diets are not known as randomized controlled trials are not practical in children.^8^ Our hypothesis is that long-term copper restriction in WD will not further improve the clinical course and liver enzymes but may result in dietary imbalance.

## Materials and Methods

This study was approved by the Non-Interventional Clinical Research Ethics Board of Hacettepe University (GO-19/415). Written informed consents were obtained from all patients and/or caregivers.

### Study Population and Data Collection

This observational study held between May 2019 and May 2020 included 32 pediatric patients with genetically confirmed WD who had been on a chelating agent plus zinc therapy at least for 12 months.

Demographic and clinical features, as well as body mass index (BMI), z-scores at disease onset were noted. Chelating agents and laboratory parameters (complete blood counts, serum liver enzymes, albumin, prothrombin time, ceruloplasmin levels, serum TCC, and UCE) were evaluated. Ceruloplasmin levels were analyzed by nephelometric assay, and the lower limit of normal was determined as 20 mg/dL.

In our institution, no sole zinc therapy is prescribed for WD. All of the patients receive a chelating agent (the first legally allowed agent is DPA and trientine is recommended in case of intolerance or non-efficacy) concomitantly with zinc. The dosage of DPA or trientine was maintained mostly between 10 and 20 mg/kg/day. The zinc formulation used in our institution was zinc sulfate. The preferred elemental zinc dosages in different ages were 50 mg/day in 2 doses (<5 years old), 75 mg/day in 3 doses (<50 kg, ≥5 years old), and 150 mg/day in 3 doses (≥50 kg). All patients receiving DPA are recommended to have vitamin B6 supplementation.

Total copper concentration was converted from “μmol/L” to “μg/dL” in order to use in the calculation of NCBC according to the equation below:

- Total copper concentration (μg/dL)^9^ = [TCC (μmol/L)]× 6.37 (reference values for TCC: 80.1-159.5 μg/dL).
  Non-ceruloplasmin-bound copper concentration is used for detecting excess levels of toxic copper and it may not be applicable in severe copper deficiency. In addition, some patients may have negative NCBC although it is not physiological. For this purpose, TCC was adjusted for ceruloplasmin to calculate serum ACC.
- Non-ceruloplasmin-bound copper concentration (μg/dL)^5^ = [TCC (μg/dL) – 3× [ceruloplasmin (mg/dL)]
  Normal limits for NCBC^5,10^: 10-15 μg/dL, excessive limit for NCBC: >25 μg/dL
- Adjusted copper concentrations (μmol/L)^11^ = [TCC(μmol/L)] – 0.052× [ceruloplasmin (mg/L)] +17.5
  Normal limits for ACC^11^: 12.7-21.5 μmol/L

The upper limit of alanine aminotransferase (ALT) was deemed as 40 IU/L according to our laboratory reference values. Patients were divided into 2 groups based on twice the upper limit of normal (ULN) ALT levels to compare their dietary copper content and median serum and urine copper parameters.

### Nutritional Assessment

Patients and/or their caregivers were asked to fill out 5-day nutritional records in order to assess daily nutrition parameters. In addition to copper and total energy consumption, macronutrients [protein (g/kg/day), percentage of energy gained from fat and carbohydrate (%), fiber (g/day)], vitamins [vitamin A (μg/day), vitamin D (μg/day), vitamin E (mg/day), vitamin K (μg/day), vitamin B1 (mg/day), vitamin B2 (mg/day), niacin (mg/1000 kcal), pantothenic acid (mg/day), vitamin B6 (mg/day), biotin (μg/day), folate (μg/day), vitamin B12 (μg/day), vitamin C (mg/day)], and particular minerals’ [zinc (mg/day), iron (mg/day)] intakes were analyzed using Bebis 7.1 Program.

The sufficiency of dietary intake of macro- and micronutrients was evaluated according to reference values of the Turkish Nutrition Guideline.^12^ Daily copper consumption (DCC) <1.3 mg/day in females and <1.6 mg/day in males was defined as “low DCC group.”^12,13^ The patients and/or their caregivers were given a questionnaire about their attitudes toward dietary copper restriction. Based on their statements, the patients were divided as either “following a copper-restricted diet” or “not restricting copper.” The normal ranges for percentage of energy gained from fat and carbohydrate were defined as 20%-35% and 45%-60%, respectively.^12^ Minimum required daily fiber intake (g/day) was calculated as “patient age (years) + 5.”

### Statistical Analysis

Statistical analyses were performed using International Business Machines Statistical Package for the Social Science Statistics, Version 23.0 for Windows (IBM Corp.; Armonk, NY, USA). If the data were normally distributed, continuous variables were expressed by the means ± standard deviations, otherwise as medians (interquartiles). Categorical variables were stated as numbers with percentages. Mean and median values of independent 2 groups were compared with Student’s *t* test and Mann–Whitney *U* test, respectively. The difference between 2 normally distributed paired samples was tested with paired sample *t*-test. Categorical variables were compared by *χ* ^2^-test or Fisher’s exact test. The relationship between 2 numerical characteristics was tested using Spearman’s test (at least 1 variable was not normally distributed). *P* value <.05 was accepted as statistically significant.

## Results

### Overall Clinical and Laboratory Features of Patients

A total of 32 patients (female/male: 17/15) were included. There were 10 siblings from 5 families in this cohort and 19 patients (59.4%) were born to consanguineous parents. Thirty patients (93.8%) presented with hepatic manifestations. Some clinical features are noted in Table 1. All patients were on chelating agents (30 patients on DPA and 2 patients on trientine hydrochloride) and concurrent zinc treatment. Although all patients receiving DPA were recommended to take vitamin B6, 8 patients (26.7%) discontinued it during follow-up. The mean daily dose of DPA and trientine hydrochloride was 16.9 ± 2.9 mg/kg (11.8-23.6). The daily dose of chelating agents was ≥20 mg/kg in 5 patients (15.6%), 15-20 mg/kg in 18 patients (56.3%), and 10-14.9 mg/kg in 9 patients (28.1%).

Laboratory tests at study enrollment showed normal hemoglobin, albumin, bilirubin levels, and prothrombin time in all patients. Alanine aminotransferase levels higher than 1×ULN were observed in 15 patients (46.9%) and 11 of them had ALT ≥ 2×ULN.

### Serum, Urine, and Dietary Copper Parameters

The median TCC, NCBC, ACC, and UCE are presented in Table 1. The TCC was low in 25 patients (78.1%). The NCBC was low, normal, and high in 17 (53.1%), 7 (21.9%), and 8 patients (25%), respectively. Seven of 17 patients with low NCBC had values below 0. Hence, we calculated ACC and all of them were within normal range except for 1 patient with high ACC. As expected, serum TCC and NCBC were strongly correlated (*P* < .001, *r* = 0.664), but none of them were correlated with UCE. In addition, ACC was correlated moderately with TCC (*P* = .020, *r* = 0.409) and strongly with NCBC (*P* < .001, *r* = 0.905) but not with UCE. Despite serum median TCC and NCBC being similar in females and males, ACC was higher in females (19.5 vs. 17.7 μmol/L, *P* = .027).

The mean DCC was 1.79 ± 0.54 mg/day (0.86-2.91). Males showed higher DCC (the mean: 2.0 vs. 1.6 mg/day, *P* = .031). In the diet query, 27 patients (84.4%) reported themselves as “following a copper-restricted diet.” However, only 6 (22.2%) of them showed low DCC. With 1 more patient who reported to be “not restricting copper,” 7 patients (21.9%) of the study group showed low DCC. Despite a mild negative correlation with TCC (*P* = .041, *r* = –0.363), DCC showed no correlation with NCBC, ACC, and UCE. The median TCC was found to be higher in the “low DCC group” than “normal/high DCC group” (*P* = .043), but NCBC, ACC, and UCE were not different. 

No correlation was detected between the serum liver enzymes and DCC. Not only DCC but also median TCC, NCBC, ACC, and UCE showed no statistical difference between the groups of “ALT <80 IU/L” vs. “ALT >80 IU/L.” The frequencies of patients with low TCC, NCBC, ACC, UCE, and DCC according to ALT threshold of 2×ULN were not different (Table 2).

### Nutritional Assessment of the Study Population

The mean BMI z-scores at the time of diagnosis and study enrollment were 0.25 ± 1.2 (–1.8 to 3.5) and –0.03 ± 1.3 (–3.47 to 3.11), respectively (*P* = .224). Twelve patients (37.5%) showed a mild decrease in their BMI z-scores. Although not statistically significant, low DCC was more common in patients with a decreased BMI z-score from diagnosis to study enrollment than steady BMI z-score group (n = 5/12, 41.7% vs. n = 2/20, 10%; *P* = .073).

Inadequate calorie intake was detected in 22 patients (68.8%). The percentage of energy from carbohydrates was lower than required in 6 patients (18.8%). Daily protein intake (g/kg/day) was high, sufficient, and insufficient in 7 (21.9%), 19 (59.4%), and 6 patients (18.8%), respectively. The percentage of energy from fat was high in 15 patients (46.9%) and 13 of them could not achieve adequate energy intake. Seven of 15 patients (46.7%) with ALT ≥40 IU/L consumed high-fat diet.

Inadequate fiber consumption was determined in 20 patients (62.5%) and 40% of them consumed fiber less than 50% of daily required. All patients with low fiber intake received insufficient total calorie.

The amounts of total energy, macro-, and micronutrients in the low and normal DCC groups were compared (Table 3). Despite no statistical significance, insufficient total calorie intake was more common in low DCC group. High fat (85.7% vs. 36%, *P* = .033), as well as inadequate fiber (100% vs. 52%, *P* = .029) and vitamin B6 (100% vs. 36%, *P* = .007), intake was more frequent in the low DCC group. Furthermore, although not statistically significant, the frequency of insufficient intake of vitamins and some minerals was higher in the low DCC group.

## Discussion

Monitoring of the WD treatment relies on indirect methods of copper detection in blood and urine as there is no precise method to estimate the total body copper amount.^14^ Although there is an inverse relationship between DCC and copper absorption under physiological conditions,^8^ this balance is impaired due to exceeded copper threshold in WD. Therefore, an additional restriction of DCC is required during WD treatment. In 1965, the experts recommended long-term strict DCC as DPA efficacy decreases over time.^15^ Recently, the American Association for the Study of Liver Diseases and the European Association for the Study of the Liver (EASL) recommend avoiding high DCC for the first 12 months of the treatment.^16^ The European Society for Pediatric Gastroenterology, Hepatology and Nutrition states that low DCC may not improve the treatment outcomes after the initiation of chelating agents. Nevertheless, avoiding high DCC until symptomatic and biochemical improvement is also advised.^17^

Based on the different nutritional and clinical features of monozygotic twins with WD, the disease is asserted to be highly related to environmental factors.^18^ We hypothesized that DCC may affect liver enzymes, as well as nutritional status. To the best of our knowledge, this is the first pediatric study to evaluate the relationship of DCC, an environmental factor, with the liver enzymes, nutritional status, and daily macro- and micronutrient consumption.

In this study group with a mean treatment duration of 85 months, 84.4% of 32 patients reported themselves as “following a copper-restricted diet,” although, in reality, only 21.9% of the patients appeared to show low DCC. Low serum TCC and NCBC were detected in 78.1% and 53.1% of the patients, respectively. Total copper concentration is expected to be reduced by treatment; however, extremely low TCC may refer to exaggerated systemic copper removal that may cause copper deficiency.^17,19^ Despite inter-individual variations, UCE and NCBC are high at diagnosis but decrease over time by treatment. While NCBC is 5%-10% of TCC in healthy controls, in WD, it is approximately 30%-50% of TCC, especially when TCC < 10 μmol/L.^19^ Although NCBC is more valuable in determining the toxicity levels of copper, extremely low NCBC may represent copper deficiency. Copper plays an essential role in cellular respiratory processes, biosynthesis of neuroendocrine enzymes, hematopoiesis, pigmentation, connective tissue formation, and body defense.^1,2^ Monitoring copper levels in WD treatment includes not only preventing copper accumulation but also controlling copper over-excretion. Copper deficiency during WD treatment may be challenging as it may result in bone marrow involvement (neutropenia, hypochromic microcytic anemia), growth retardation, hair hypopigmentation, and predisposition to infections.^1^ Despite the high frequency of reduced TCC and NCBC in our study population, no complication related to copper depletion was detected. Interestingly, ACC, another adjusted copper parameter, was normal in all except 1 patient, unlike NCBC. Hence, this finding may support the use of ACC instead of NCBC to estimate the excessively reduced body copper.

In our study, DCC was inversely correlated with TCC (*P* = .041, *r* = –0.363). The median TCC was found to be higher in the “low DCC group” (*P* = .043), while NCBC, ACC, and UCE were not different. The inverse relationship shown between DCC and TCC may suggest a probability of a preventive process of the copper mechanism during long-term treatment as in the physiological state. However, physiological behavior of copper absorption is unexpected as copper excretion is still high during WD treatment. Hence, the effect of DCC on 2 parameters, NCBC and ACC, which represent toxic or extremely low copper levels, is more reasonable to show the relationship between diet and copper parameters. Our study revealed the lack of association between DCC and NCBC and ACC.

In our study, males showed higher DCC, as expected. Copper absorption may also be affected by age, sex, type of foods, and drugs.^8^ Copper levels that are adjusted for ceruloplasmin gave the opportunity to overcome some variables (sex, age, adequacy of measuring method) that affect ceruloplasmin.^11^ After adjusting TCC as ACC, we observed that females showed higher ACC. This difference still may be due to hormonal variations^20^ despite adjustment or due to different severity of the disease histologically.^17^

Genetic and epigenetic factors determine the severity of WD. Some factors such as DCC, antioxidant capacity, hormonal effects, susceptibility to liver fibrosis, and timing of the liver injury may vary among WD patients.^6^ Due to the prolonged dietary restriction of copper in WD, we evaluated the relationship between DCC and high ALT. Our study population did not show a difference in mean DCC between groups with ALT <80 IU/L vs. ≥80 IU/L. Hence, we speculate that there may be no additional effect of prolonged copper restriction on ALT levels. Besides, serum and urine copper parameters in these 2 ALT groups were similar. With these results, we argued that elevated ALT levels may not always represent non-compliance or serum and urine copper parameters may miss some treatment non-compliance.

Insufficient energy intake was determined in 68.8% of the study population and could not be overcome despite high-fat contents in 59.1% (n = 13) of them. Protein-containing foods are rich in copper and our study showed a higher, but not significant, ratio of low protein intake in the low DCC group. Liver, mushrooms, nuts, legumes, dried fruits, multigrain bread, shellfish, and chocolate are the foods mostly recommended to avoid in copper-restricted diets.^2,8^ Most of these foods, except for liver and shellfish, require an excessive amount to reach the upper limit for copper in WD. Thus, solely liver and shellfish avoidance is still recommended in the long term.^10^ However, shellfish consumption is rare in Turkish children, and copper restriction can be performed by mostly reducing meat and legume consumption which causes lower protein intake. In the English abstract of the Russian-written study from Baranovsky et al^21^, it was stated that protein-modified diets (20 g/day of dry composite protein mixture) in cirrhotic and non-cirrhotic WD patients resulted in improvement not only in anthropometric parameters but also in bilirubin and free copper levels. Patients with low protein intake probably tried to compensate it by high-fat consumption, so the low DCC group showed significantly high fat consumption (*P* = .033). However, high fat consumption did not compensate for adequate calorie intake. Hence, despite no significance, low DCC group showed more frequent insufficient calorie intake than the normal DCC group. A decrease in BMI z-score was determined in 12 patients (37.5%) and low DCC was more common in patients with decreased BMI z-score but not statistically significant.

While evaluating the association of ALT and fat content of the diet, we found that 7 of 15 patients (46.7%) with high ALT consumed fat-rich diet which is a risk for steatohepatitis. A study in rats showed that high calorie intake resulted in earlier liver damage accompanied by mitochondrial structural injury.^18^ To and Schilsky^22^ suggested that dietary recommendations may be changed in close future to reduce the hepatic damage in WD, not only a low-fructose but also low-fat diet could be suggested with decoppering drugs.

In WD, it has been suggested that lacto-vegetarian diets reduce copper bioavailability due to high fiber and phytate content.^8,23^ However, lack of fiber consumption was more common in the low DCC group (100% vs. 52%, *P* = .029). This finding underlines the importance of evaluating fiber content of the copper-restricted diet. All patients with low fiber intake received low energy that may have resulted from avoiding multigrain bread, legumes, and dried fruits. So, patients and caregivers should be educated about fiber adequacy and other sources of fiber.

The low DCC group showed insignificant but higher ratios of insufficiency of all vitamins, especially water-soluble vitamins. Inadequate vitamin B6 consumption was significantly more frequent in low DCC group (*P* = .007). With this finding, we suggest that not only side effect of DPA but also dietary low intake may contribute to vitamin B6 deficiency during WD treatment. Enrichment of the diet with micronutrients, especially during copper-restricted diet, is essential as some multivitamins include copper.^8^

There are some limitations to this study. The study population was small as this study was designed in 1 institution. Due to the severe progression of the disease and institutional policy for WD, avoiding dietary copper restriction during WD’s drug treatment or recommending only copper restriction without DPA or trientine cannot be considered; therefore, the study design could not be designed as a randomized control study. However, a prospectively designed study comparing patients with and without copper restriction during WD treatment would clarify the effect of the diet. According to our institutional policy, all patients received a chelating agent as well as zinc treatment concomitantly. Therefore, the use of these medications may have strongly and variably affected the levels of blood and urine copper parameters. Patients reported treatment adherence, but verbal statements may not reflect the reality. Urine and serum copper parameters obtained in a single visit may be insufficient to directly identify the patients with non-adherence so recurrent measurements are needed.

In conclusion, DCC may have the least or no effect on liver enzymes in WD. The recommendation of copper restriction may cause disease-related anxiety, unintentional avoidance of essential nutrients, and eventually result in malnutrition. A long-term low DCC, except for liver and shellfish consumption, may not contribute to the treatment efficacy. However, nutritional assessment should be a part of WD monitoring. This approach may not only prevent undernutrition but also circumvent high-calorie and fat-containing diets in order to reduce the risk of hepatic steatosis. Enrichment of the diet with vitamins, especially vitamin B6, as well as with fiber, should be encouraged in WD patients, regardless of DCC.

## Figures and Tables

**Table 1.** The Clinical Features and Copper Parameters of the Study Population

Variable	Results
Age at disease-onset, years, mean ± SD (range)	7.1 ± 3.7 (1.6-16)
Age at study enrolment, years, mean ± SD (range)	14.2 ± 3.2 (6.6-18)
Duration of follow-up, months, mean ± SD (range)	85.8 ± 51.8 (20-199)
TCC, μg/dL, median (IQR)	22.1 (65.8)
NCBC, μg/dL, median (IQR)	7.05 (16.7)
ACC, μmol/L, median (IQR)	18.3 (2.5)
UCE, μg/24 h, median (IQR)	383.5 (302.3)

ACC, adjusted copper concentrations (reference values: 12.7-21.5 μmol/L); IQR, interquartile range; NCBC, non-ceruloplasmin bound copper concentrations (reference values: 10-15 μg/dL); SD, standard deviations; TCC, serum total copper concentrations (reference values: 80.1-159.5 μg/dL); UCE, 24-hour urine copper excretion.

**Table 2.** The Comparison of Copper-related Parameters in 2 Groups Defined According to Twice the Upper Limit of Normal Alanine Aminotransferase

	ALT< 80 IU/L (n = 21)	ALT ≥ 80 IU/L (n = 11)	*P*
TCC, μg/dL, median (IQR)	30.3 (72.0)	17.3 (26.7)	.137
Patients with low TCC, n (%)	15 (71.4)	10 (90.9)	.374
NCBC, μg/dL, median (IQR)	6.5 (19.2)	7.6 (14.2)	.766
Patients with low NCBC, n (%)	11 (52.4)	6 (54.5)	.925
ACC, μmol/L, median (IQR)	18.2 (2.6)	18.3 (2.4)	.592
UCE, μg/24 h, median (IQR)	384 (302)	285 (347)	.766
Patients with UCE < 250 μg/24 h, n (%)	6 (28.6)	4 (36.4)	.703
DCC, mg/day, mean ± SD	1.8 ± 0.6	1.7 (0.5)	.485
Patients with low DCC^1^, n (%)	5 (23.8)	2 (18.2)	1.0

^1^Daily copper consumption lower than 1.3 mg/day for females and 1.6 mg/day for males

ACC, adjusted copper concentrations; ALT, alanine aminotransferase; DCC, daily copper consumption; IQR, interquartile range; NCBC, non-ceruloplasmin bound copper concentrations; SD, standard deviations; TCC, total copper concentrations; UCE, 24-hour urine copper excretion.

**Table 3.** Nutritional Features of the Patients with Wilson Disease According to Daily Copper Consumption

		Low DCC^1^	Normal DCC	*P*
Energy intake	Insufficient	7 (100)	15 (60)	.069
	Sufficient	0	10 (40)	
Protein consumption	Insufficient	2 (28.6)	4 (16)	.590
	Sufficient and high	5 (71.4)	21 (84)	
Fat consumption	Sufficient	1 (14.3)	16 (64)	.033*
	High	6 (85.7)	9 (36)	
Carbohydrate consumption	Insufficient	3 (42.9)	3 (12)	.101
	Sufficient	4 (57.1)	22 (88)	
Fiber intake	Insufficient	7 (100)	13 (52)	.029*
	Sufficient	0	12 (48)	
Vitamin A	Insufficient	3 (42.9)	7 (28)	.648
	Sufficient and high	4 (57.1)	18 (72)	
Vitamin E	Insufficient	2 (28.6)	5 (20)	.632
	Sufficient	5 (71.4)	20 (80)	
Vitamin B1	Insufficient	7 (100)	21 (84)	.552
	Sufficient	0	4 (16)	
Vitamin B2	Insufficient	6 (85.7)	10 (40)	.083
	Sufficient	1 (14.3)	15 (60)	
Vitamin B3	Insufficient	7 (100)	19 (76)	.296
	Sufficient	0	6 (24)	
Vitamin B6	Insufficient	7 (100)	9 (36)	.007*
	Sufficient	0	16 (64)	
Vitamin B12	Insufficient	6 (85.7)	12 (48)	.104
	Sufficient	1 (14.3)	13 (52)	
Vitamin C	Insufficient	4 (57.1)	9 (36)	.401
	Sufficient	3 (42.9)	16 (64)	
Pantothenic acid	Insufficient	7 (100)	16 (64)	.149
	Sufficient	0	9 (36)	
Biotin	Insufficient	6 (85.7)	13 (52)	.195
	Sufficient	1 (14.3)	12 (48)	
Folate	Insufficient	6 (85.7)	17 (68)	.640
	Sufficient	1 (14.3)	8 (32)	

*P ≤ .05 is statistically significant.

The numbers are expressed in numbers (%).

^1^Low DCC, daily copper consumption lower than 1.3 mg/day for females and 1.6 mg/day for males.

DCC, daily copper consumption.
